# Supplementary material for: Deletion or Inhibition of Astrocytic Transglutaminase 2 Promotes Functional Recovery after Spinal Cord Injury
Source: Cells. 2021 Oct 29;10(11):2942. doi: 10.3390/cells10112942 (PMC8616117; doi:10.3390/cells10112942)
Supplement: Supplementary file 1 [file cells-10-02942-s001.zip › cells-1382972-supplementary.pdf]

Supplemental Figure S1

Supplementary Code 1 & 2.ijm

```
//These are the variables to change for the threhold cell counts.
//These are the variables used for the NeuN cell count.
//They real values must be empirically determined by user.


lowT = 0;
upperT = 70;
lowerS= 25;
upperS= 2000;



dir = getDirectory("choose a Directory")
list = getFileList(dir);

Dialog.create("threshold");
Dialog.addNumber("Lower", lowT);
Dialog.addNumber("Upper", upperT);
Dialog.show();
//setThreshold(lowT, upperT);

for (i = 0; i < list.length; i++){
   if(File.isDirectory(dir+list[i])){}
   else {
   open(dir+list[i]);
  //run("Split Channels");
   //close();
   //close("\\Others");

   getDimensions(width, height, channels, slices, frames);
   title = File.nameWithoutExtension;
   if (slices > 1){
    run("Z Project..."); //error add code max int
    }
    setThreshold(lowT, upperT);
    run("Threshold");
    run("Invert");
    run("Analyze Particles...", "size="+ lowerS + "-" + upperS + " show=Overlay summarize in_situ include_holes");
    run("Close All");
    }
}
```

```
//These are the variables used to measure GFAP fluorescence intensity.
//They real values must be empirically determined by user

makeRectangle(12, 18, 2736, 2562);
waitForUser;
run("Crop");
setThreshold(0, 80);
run("Create Selection");
run("Measure"); // Make sure you have area integrated intensity and mean grey value selected
```

Supplemental code for Fiji Image analysis used for immunofluorescence quantification.

Supplemental Figure S2

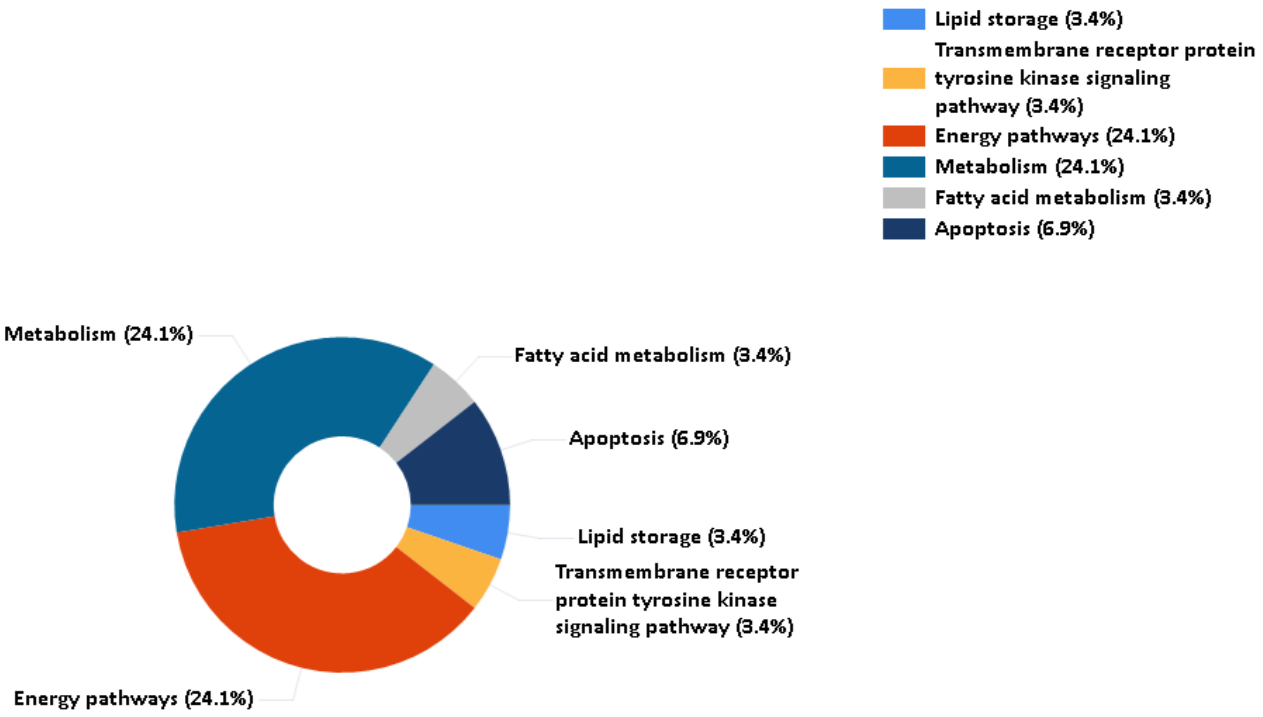


Functional analysis of genes that are significantly upregulated in GFAP-Cre+/-TG2fl/fl mice three days post injury using FunRich (http://www.funrich.org).

## Supplemental Table S1

| ANTIBODY | SOURCE | CATALOGUE NUMBER |
|---|---|---|
| rabbit anti-GFAP | Dako | Z033401-2 |
| goat anti-GFAP | Novus | NB100-53809 |
| rabbit anti-SOX9 | Millipore-Sigma | AB5535 |
| chicken anti-NeuN | Millipore-Sigma | ABN91 |
| Anti-NG2, Alexa Fluor 488 conjugate | Millipore-Sigma | AB5320A4 |
| donkey anti-goat Alexa Fluor 488 | Invitrogen | A-11055 |
| goat anti-chicken Alexa Fluor 488 | Invitrogen | A32931 |
| goat anti-rabbit Alexa Fluor 647 | Invitrogen | A32733 |
| donkey anti-goat Alexa Fluor 647 | Invitrogen | A32849 |
| donkey anti-rabbit Alexa Fluor 647 | Invitrogen | A48258 |

## Supplemental Table S2

| Target | Forward primer | Reverse primer |
|---|---|---|
| Lipoprotein lipase | 5’GGGAGTTTGGCTCCAGAGTTT3′ | 5’TGTGTCTTCAGGGGTCCTTAG3’ |
| Fatty acid binding protein 4 | 5’AAGGTGAAGAGCATCATAACCCT3’ | 5’TCACGCCTTTCATAACACATTCC3’ |
| Perilipin | 5’GGGACCTGTGAGTGCTTCC3’ | 5’GTATTGAAGAGCCGGGATCTTTT3’ |
| Glyceraldehyde-3-Phosphate Dehydrogenase | 5’ATGGGACGATGCTGGTACTAG3’ | 5’TGCTGACAACCTTGAGTGAAAT3’ |

Supplemental Table S3

**Genes that are signficantly different in spinal cords of GFAP-Cre+/-TG2fl/fl mice compared to TG2fl/fl mice 3 days postinjury**

| Gene | BaseMean | log2FoldChange | stat | pvalue | padj |
|---|---|---|---|---|---|
| Ucp1 | 1839.018 | 3.204 | 7.039 | 1.94E-12 | 5.16E-08 |
| Pck1 | 544.603 | 3.23 | 6.864 | 6.69E-12 | 8.91E-08 |
| Cidec | 379.985 | 3.29 | 6.378 | 1.80E-10 | 1.60E-06 |
| Plin1 | 229.471 | 3.241 | 6.119 | 9.43E-10 | 6.29E-06 |
| Adipoq | 165.857 | 3.359 | 6.066 | 1.31E-09 | 7.01E-06 |
| Tmem79 | 76.832 | 2.268 | 5.739 | 9.53E-09 | 4.23E-05 |
| Trarg1 | 313.398 | 2.7 | 5.27 | 1.37E-07 | 0.00052 |
| Tmem45b | 47.06 | 2.373 | 5.203 | 1.97E-07 | 0.000655 |
| Hp | 2084.877 | 3.129 | 5.149 | 2.62E-07 | 0.000777 |
| Ces1d | 49.255 | 2.407 | 5.07 | 3.98E-07 | 0.001062 |
| Cidea | 585.054 | 2.679 | 4.897 | 9.72E-07 | 0.002355 |
| Retn | 23.804 | 2.775 | 4.865 | 1.14E-06 | 0.002539 |
| Fabp4 | 4711.471 | 2.878 | 4.843 | 1.28E-06 | 0.002621 |
| Cd300lg | 111.464 | 2.537 | 4.777 | 1.78E-06 | 0.003165 |
| Adtrp | 82.11 | 2.817 | 4.789 | 1.67E-06 | 0.003165 |
| Lpl | 2917.602 | 2.565 | 4.753 | 2.00E-06 | 0.003334 |
| Btnl9 | 31.183 | 2.496 | 4.611 | 4.01E-06 | 0.006283 |
| Aqp7 | 47.057 | 2.51 | 4.584 | 4.57E-06 | 0.006761 |
| Elovl3 | 157.437 | 2.352 | 4.495 | 6.94E-06 | 0.009743 |
| Th | 109.153 | 1.704 | 4.433 | 9.29E-06 | 0.012381 |
| Ctcflos | 55.689 | 2.004 | 4.417 | 1.00E-05 | 0.012714 |
| Cyp2e1 | 67.719 | 2.278 | 4.288 | 1.81E-05 | 0.021228 |
| Cfd | 219.85 | 2.35 | 4.285 | 1.83E-05 | 0.021228 |
| Adig | 27.797 | 2.314 | 4.244 | 2.20E-05 | 0.024394 |
| Ntrk1 | 62.048 | 1.941 | 4.216 | 2.49E-05 | 0.02455 |
| Otop1 | 58.498 | 2.495 | 4.224 | 2.40E-05 | 0.02455 |
| Pirt | 238.056 | 2.063 | 4.218 | 2.47E-05 | 0.02455 |
| Acaa1b | 104.13 | 2.317 | 4.154 | 3.27E-05 | 0.031167 |
| Alkal2 | 19.039 | 1.863 | 4.141 | 3.46E-05 | 0.031789 |
| Slc6a2 | 25.009 | 2.4 | 4.072 | 4.66E-05 | 0.041444 |
| Slc18a2 | 18.511 | 2.269 | 4.063 | 4.85E-05 | 0.041741 |
| Cyp2f2 | 10.428 | 2.267 | 4.032 | 5.54E-05 | 0.045051 |
| C7 | 19.398 | 2.371 | 4.03 | 5.58E-05 | 0.045051 |
| Chad | 48.389 | 1.949 | 4.012 | 6.01E-05 | 0.047116 |
| Acot2 | 246.088 | 1.395 | 3.989 | 6.64E-05 | 0.049205 |
| 2010003K1 | 17.845 | 2.458 | 3.989 | 6.63E-05 | 0.049205 |
| Ccdc80 | 318.311 | 1.054 | 3.98 | 6.89E-05 | 0.049669 |

Supplemental Table S4

| Biological Process | Adjusted P-v | Odds Ratio | Combined Score | Genes |
|---|---|---|---|---|
| brown fat cell differentiation (GO:0050873) | 1.52E-04 | 352.2 | 5262.453429 | ADIPOQ;UCP1;ADIG |
| long-chain fatty acid metabolic process (GO:0001676) | 0.00268941 | 30.50863061 | 336.1408856 | ELOVL3;ACOT2;ADTRP;CYP2E1 |
| response to insulin (GO:0032868) | 0.00268941 | 30.12575758 | 330.4923304 | TRARG1;ADIPOQ;OTOP1;PCK1 |
| positive regulation of cold-induced thermogenesis (GO:0120162) | 0.00356049 | 25.89768654 | 269.3920495 | FABP4;ADIPOQ;ELOVL3;UCP1 |
| cellular response to peptide hormone stimulus (GO:0071375) | 0.00403567 | 23.60190137 | 237.2875724 | TRARG1;ADIPOQ;OTOP1;PCK1 |
| positive regulation of metabolic process (GO:0009893) | 0.00431888 | 22.07839867 | 216.4478246 | FABP4;ADIPOQ;ELOVL3;UCP1 |
| synaptic transmission, dopaminergic (GO:0001963) | 0.00465807 | 228.0914286 | 2183.712167 | TH;SLC6A2 |
| cellular response to insulin stimulus (GO:0032869) | 0.00474921 | 19.23684848 | 178.6773171 | TRARG1;ADIPOQ;OTOP1;PCK1 |
| monoamine transport (GO:0015844) | 0.00474921 | 190.0666667 | 1765.211478 | SLC6A2;SLC18A2 |
| response to glucose (GO:0009749) | 0.00474921 | 38.20396419 | 351.3922501 | ADIPOQ;LPL;PCK1 |
| positive regulation of sequestering of triglyceride (GO:0010890) | 0.00506972 | 162.9061224 | 1472.211648 | CIDEA;LPL |
| fat cell differentiation (GO:0045444) | 0.00653855 | 31.93796791 | 277.6550177 | ADIPOQ;UCP1;ADIG |
| regulation of biosynthetic process (GO:0009889) | 0.00653855 | 126.6920635 | 1091.539977 | UCP1;PCK1 |
| fatty acid biosynthetic process (GO:0006633) | 0.0102331 | 25.81531142 | 208.9406809 | ELOVL3;LPL;CYP2E1 |
| regulation of sequestering of triglyceride (GO:0010889) | 0.01076804 | 87.69230769 | 699.2344145 | CIDEA;LPL |
| regulation of fat cell differentiation (GO:0045598) | 0.01227769 | 22.78762414 | 176.4266626 | ADIPOQ;LPL;ADIG |
| cellular response to fatty acid (GO:0071398) | 0.01227769 | 75.99238095 | 586.4606264 | UCP1;LPL |
| response to fatty acid (GO:0070542) | 0.01454584 | 67.04537815 | 502.2155781 | UCP1;LPL |
| positive regulation of lipid storage (GO:0010884) | 0.01688376 | 59.98195489 | 437.1224949 | CIDEA;LPL |
| lipid droplet organization (GO:0034389) | 0.017623 | 56.98 | 409.8811226 | CIDEA;CIDEC |
| triglyceride catabolic process (GO:0019433) | 0.01836088 | 54.26394558 | 385.4700929 | FABP4;LPL |
| response to hexose (GO:0009746) | 0.02073383 | 49.54037267 | 343.5896724 | ADIPOQ;LPL |
| negative regulation of protein transport (GO:0051224) | 0.02146006 | 47.47380952 | 325.5122514 | ADIPOQ;ADTRP |
| regulation of reactive oxygen species biosynthetic process (GO:19( | 0.0238641 | 43.81758242 | 293.9252254 | UCP1;SLC18A2 |
| regulation of macrophage derived foam cell differentiation (GO:0( | 0.02691638 | 39.27881773 | 255.4802892 | ADIPOQ;LPL |
| fatty acid metabolic process (GO:0006631) | 0.02691638 | 14.4691298 | 93.61217318 | ADIPOQ;ACOT2;LPL |
| regulation of tumor necrosis factor production (GO:0032680) | 0.02691638 | 14.4691298 | 93.61217318 | ADIPOQ;CIDEA;LPL |
| very long-chain fatty acid metabolic process (GO:0000038) | 0.02840625 | 36.74101382 | 234.3909225 | ELOVL3;ACOT2 |
| acylglycerol catabolic process (GO:0046464) | 0.02980736 | 34.51082251 | 216.1207577 | FABP4;LPL |
| regulation of lipid biosynthetic process (GO:0046890) | 0.02980736 | 34.51082251 | 216.1207577 | ADIPOQ;PCK1 |
| proton transmembrane transport (GO:1902600) | 0.03221294 | 32.5355102 | 200.1585487 | UCP1;OTOP1 |
| negative regulation of cell-cell adhesion (GO:0022408) | 0.03708499 | 29.19267399 | 173.6566538 | ADIPOQ;ADTRP |
| hexose metabolic process (GO:0019318) | 0.03708499 | 29.19267399 | 173.6566538 | ADIPOQ;PCK1 |
| negative regulation of tumor necrosis factor production (GO:0032 | 0.04516859 | 25.86883117 | 148.0109909 | ADIPOQ;CIDEA |
| negative regulation of cold-induced thermogenesis (GO:0120163) | 0.04577499 | 25.29269841 | 143.6441165 | ADIPOQ;CIDEA |
| negative regulation of tumor necrosis factor superfamily cytokine | 0.04638449 | 24.74161491 | 139.4901066 | ADIPOQ;CIDEA |
| positive regulation of multicellular organismal process (GO:00512 | 0.04649415 | 6.974851151 | 39.11575762 | FABP4;ADIPOQ;ELOVL3;UCP1 |
| positive regulation of fat cell differentiation (GO:0045600) | 0.04822795 | 23.22332362 | 128.1662168 | LPL;ADIG |
| inositol lipid-mediated signaling (GO:0048017) | 0.04822795 | 23.22332362 | 128.1662168 | NTRK1;PIRT |

**Gene ontology pathway analysis identifies a preponderance of lipid biosynthetic pathways among differentially expressed genes in mice lacking astrocytic TG2.** 22 out of 39 GO terms with an adjusted p-value of less than 0.05 are involved with lipid biosynthetic pathways and storage (highlighted in yellow). Enrichr, GO Biological Process 2021.
